# Oxidative Stress and Salivary Physicochemical Characteristics Relative to Dental Caries and Restorative Treatment in Children

**DOI:** 10.3390/antiox14040405

**Published:** 2025-03-28

**Authors:** Anastasia A. Poimenidou, Panagiota Geraki, Sotiria Davidopoulou, Sotirios Kalfas, Aristidis Arhakis

**Affiliations:** 1 Department of Paediatric Dentistry, Aristotle University of Thessaloniki, 54124 Thessaloniki, Greece; giotageraki@gmail.com (P.G.); arhakis@dent.auth.gr (A.A.); 2 Department of Operative Dentistry, Aristotle University of Thessaloniki, 54124 Thessaloniki, Greece; sdavidop@dent.auth.gr; 3 Department of Preventive Dentistry, Periodontology and Implant Biology, Aristotle University of Thessaloniki, 54124 Thessaloniki, Greece

**Keywords:** saliva, flow rate, conductivity, buffer capacity, pH, dental caries, oxidative stress, 8-Epi-prostagladin F2 alpha, 8-isoprostane, dental treatment

## Abstract

Numerous studies investigating oxidative stress in saliva in relation to dental caries and dental treatment focus on parameters such as total antioxidant capacity and malondialdehyde. This study is the first to utilize 8-isoprostane as a salivary biomarker to assess oxidative stress in relation to both caries and dental treatment. Salivary properties are also evaluated. The innovation of this study lies in its unique approach: although these salivary parameters have been examined individually in past research, this is the first study to evaluate them in conjunction with oxidative stress. A total of 26 children with caries and 20 caries-free children aged between 4 and 12 years old were included in this study. From both groups, stimulated saliva was collected and used to assess salivary properties and the 8-isoprostane level. For the caries group, the same measurements were also conducted after dental treatment. Children with caries had significantly lower levels of pH, and conductivity compared to caries-free children. The concentration of 8-isoprostane did not differ significantly between the two groups. In the caries group, dental treatment led to an increase in salivary conductivity and buffer capacity, along with a decrease in 8-isoprostane levels.

## 1. Introduction

The term “dental caries” refers to the visible signs and symptoms resulting from a localized chemical dissolution. This occurs due to changes in bacterial activity and the environment within the biofilm covering the affected tooth [1]. Microbial shifts are thought to increase acid production in dental plaque, disrupting the balance between demineralization and remineralization, which, in turn, promotes mineral loss and accelerates the development of caries [2].

Saliva plays a crucial role in the homeostasis of the oral environment [3]. Among the functions that contribute to caries prevention are the clearance of food debris and microbial end products from the tooth surfaces, the supply of calcium and phosphate ions that enable the remineralization of carious lesions, and pH stabilization around the neutral level through its bicarbonate and phosphate buffer systems [3,4]. When the salivary flow decreases, its buffering capacity becomes less effective, reducing its ability to neutralize acids and increasing the likelihood of caries developing [1]. Unlike the well-established link between salivary flow rate and caries risk, the relationship between dental caries, salivary buffer capacity, and pH remains less conclusive [5].

Saliva is a valuable diagnostic fluid for detecting systemic diseases and oral inflammatory conditions, including dental caries, due to its easy and non-invasive collection [6]. In addition to serving as a marker for various conditions, saliva can also be utilized to assess levels of oxidative stress [7]. Oxidative stress occurs when there is an imbalance between the production of free radicals, such as reactive oxygen species (ROS), and the body’s antioxidant defense mechanisms, both enzymatic and non-enzymatic [8]. Many studies examining oxidative stress in saliva focus on parameters like total antioxidant capacity (TAC) and malondialdehyde (MDA), which are byproducts of lipid peroxidation resulting from oxidative stress [7,9]. TAC is commonly used as a marker of saliva’s antioxidative capacity, while MDA reflects oxidative damage [7]. Additional biomarkers, such as uric acid and superoxide dismutase (SOD), are also used to assess oxidative stress levels [10].

In addition to the above biomarkers, isoprostanes serve as reliable markers of oxidative stress, characterized by their chemical stability and specificity as byproducts of lipid peroxidation. Research has identified notably higher levels of salivary 8-Epi-prostaglandin F2 alpha, also known as 8-isoprostane, in individuals with oral submucous fibrosis and oral squamous cell carcinoma compared to healthy subjects [11]. Similarly, elevated levels have been detected in cases of oral lichen planus, particularly in the erosive variant [12]. Additionally, patients with chronic periodontitis, especially those with type II diabetes, have exhibited significantly raised salivary 8-isoprostane levels compared to healthy individuals [13]. Uncontrolled diabetic patients also demonstrated higher levels of salivary 8-isoprostane compared to controlled and non-diabetic individuals [14].

While TAC [10,15,16] and MDA [7,8,17] have been analyzed as biomarkers of oxidative stress in relation to dental caries, 8-isoprostane has not been studied in this regard, and there are no previous studies to our knowledge regarding salivary 8-isoprostane oxidative stress levels and caries either in children or adults. No relationship has yet been reported between the physico-chemical properties of saliva and salivary levels of 8-isoprostane. With these data in mind, the present study was conducted to determine the salivary levels of 8-Epi-prostaglandin F2 alpha in samples from caries-active children before and after dental restorative treatment, and in caries-free children as well. Furthermore, salivary characteristics such as flow rate, pH, buffering capacity, and conductivity are examined in the same samples and explored for potential correlations with the oxidative stress level and the dental treatment.

## 2. Materials and Methods

### 2.1. Ethical Approval

The Ethics Committee of the Dental School of Aristotle University of Thessaloniki, Greece, approved the study protocol (No. 103/2021). Informed consent was obtained from the guardians of all children, as required.

### 2.2. Participants

A total of 46 children, 26 of whom were caries-active, and 20 of whom were caries-free, aged 4 to 12 years and attending the pediatric dental clinic at Aristotle University of Thessaloniki from January 2023 to December 2023, participated in the study. The characteristics of the participants are depicted in Table 1. Two calibrated dentists examined the children using the WHO caries assessment form to determine the DMFS/dmfs and DMFT/dmft scores [18]. The calibration process involved theoretical training, practical exercises, and inter-examiner reliability testing [18]. The children included as caries-active exhibited at least five decayed tooth surfaces that required restoration, while those with a DMFS/dmfs score of 0 were placed in the caries-free group [18]. The patients included were systemically healthy, took no medication, and had no special needs. Children who were not willing to comply with the study were not enrolled. The assessment of health status and medication use was based on medical history records completed by the participant’s parents/caregivers.

### 2.3. Saliva Sampling

Stimulated saliva samples were collected from all participants. For the caries-active group, sampling was performed after gingivitis treatment (scaling and personal plaque control), before the start of the restorative treatment and one week after its completion, while for the caries-free group, sampling took place after gingivitis treatment [19]. To minimize possible variations due to circadian rhythm, sampling sessions were limited to the hours between 10:00 and 11:30 a.m. following a 2 h fasting period [20]. Children were asked to drink 0.5 L of water one hour before sampling [21]. To attain maximum cooperation on the part of the children, we replaced the paraffin wax with sugar-free xylitol-containing chewing gum. To remove the flavoring agents from the gum, the children were instructed to chew for 2 min. The sampling session started 20 min later. For this purpose, the children were asked to chew the gum again, and swallow the saliva collected in their mouth for 1 min to soften the unflavored chewing gum, and after that, saliva was collected for 5 min. The collected saliva was immediately utilized to estimate the salivary flow rate, pH, buffer capacity, and conductivity. The salivary flow rate was calculated from the total volume of the saliva sample (mL/min). A portable digital meter (HI9811-5 pH/EC/TDS/°C, HANNA instruments, Smithfield, RI, USA) was used to measure the pH and conductivity of the samples. Buffer capacity was determined using the Ericson method with the digital pH meter [22]. The samples were immediately stored at −80 °C until analysis for 8-isoprostane. The saliva samples were thawed and cleared by centrifugation at 10,000× *g* for 5 min before the ELISA analysis, which was performed with a commercially available ELISA kit (Cloud-Clone Corp; Houston, TX, USA), with high sensitivity and excellent specificity with a detection range of 24.69–2000 pg/mL.

### 2.4. Statistical Analysis

Data for the examined parameters (salivary flow rate, conductivity, pH, buffer capacity, 8-isoprostane) are presented as the min, median, max, and mean ± standard deviation plus the corresponding 95% confidence intervals around the mean values. For statistical comparisons between the two groups, Student’s *t*-test for independent samples was performed to determine the conductivity and pH parameters. For the parameters of salivary flow rate, buffer capacity, and 8-isoprostane, the Mann–Whitney test was performed for the comparison of the two groups. For the parameters of salivary flow rate, conductivity, and pH, comparative analysis in the caries of active children was completed before and after treatment with Student’s *t*-test for paired samples. For the parameter’s buffer capacity and 8-isoprostane levels, the Wilcoxon test was used to test the differences before and after treatment. In all statistical comparisons, an estimate of the corresponding effect size (Cohen’s *d* for parametric tests or index *r* for non-parametric tests) was estimated [23]. Correlations between variables were examined using Pearson’s (*r*) linear correlation coefficient and Spearman’s *rho* rank correlation coefficient.

For all examined parameters (salivary flow rate, conductivity, pH, buffer capacity, and 8-isoprostane), preliminary analyses for normality within each of the two groups were performed. Specifically, visual examinations of the corresponding histograms were not so informative since the number of measurements in each group was small for testing the fit in a distribution. Next, the values of means and medians were compared, and no important differences were found. Following this, the values of coefficients of Skewness and Kurtosis of the corresponding distributions were estimated and assessed. In most cases, the coefficients were within the limits of [−1, 1]. Finally, a series of Shapiro–Wilk tests revealed that within each group, there were doubts relative to the normality of the parameters of the salivary flow rate, buffer capacity, and 8-isoprostane (the corresponding *p*-values ranged from 0.001 to 0.037). For these three parameters, the Mann–Whitney test was used for the comparison of the two groups. The same tests were used to test the normality of the differences between the measured parameters before and after treatment. There were doubts about the normality of the differences between the parameters buffer capacity and 8-isoprostane (the corresponding *p*-values of the Shapiro–Wilk test were both <0.001). For these two parameters, the Wilcoxon test was used to test the differences before and after treatment.

In all non-parametric tests (Mann–Whitney and Wilcoxon test) the observed significance level (*p*-value) was estimated with the Monte-Carlo simulation method, utilizing 10.000 resampling circles. With this method, the inferential conclusions were safe and valid even in cases where the methodological assumptions of the non-parametric tests were not met [24].

The significance level of all statistical tests was predetermined at *a* = 0.05 (*p* ≤ 0.05). All data were processed with the IBM SPSS Statistics software (version 28.0) enhanced with module Exact Tests for implementing the Monte-Carlo simulation method.

## 3. Results

The characteristics of the participants are presented in Table 1. The physicochemical properties and oxidative stress of saliva are presented in Table 2. Compared to the caries-free children, those with active caries lesions had significantly lower levels of pH and salivary conductivity (Table 2 and Table 3). The concentration of the oxidative marker, 8-isoprostane, did not differ significantly between the two groups (Table 2 and Table 3). The physicochemical properties and oxidative stress of saliva for the caries-active group, before and after dental treatment, are presented in Table 4. There was a significant increase in the salivary conductivity and buffering capacity after dental treatment and a decrease in the concentration of 8-isoprostane (Table 4 and Table 5).

The correlations between different parameters and experiences of caries (DT+ dt) in all participants (*n* = 46) are presented in Table 6. The salivary flow rate is positively correlated with pH (Pearson correlation coefficient *r* = 0.286, *p* = 0.05, Spearman’s *rho* = 0.23, *p* = 0.124) and conductivity (Pearson correlation coefficient *r* = 0.674, *p* < 0.001, Spearman’s *rho* = 0.586, *p* < 0.001). pH correlated positively with conductivity (Pearson correlation coefficient *r* = 0.370, *p* = 0.011, Spearman’s *rho* = 0.371, *p* = 0.011) and buffer capacity (Pearson correlation coefficient *r* = 0.381, *p* = 0.009, Spearman’s *rho* = 0.418, *p* = 0.004).

**Figure 1:**
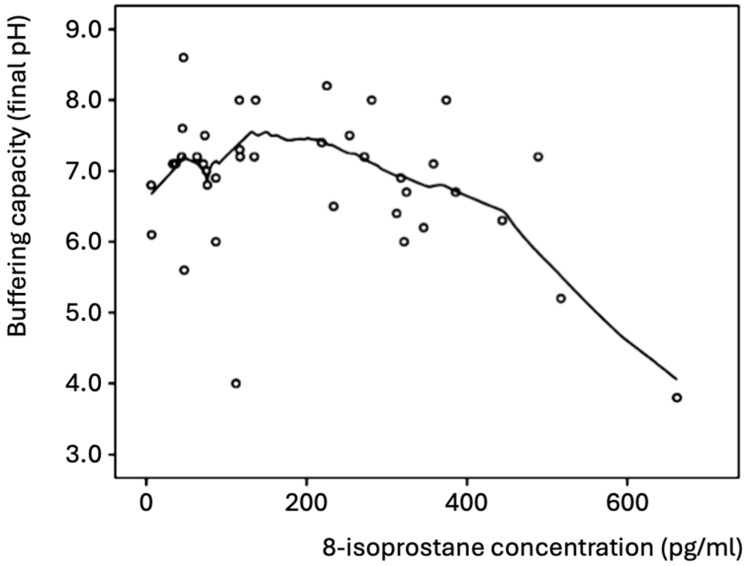
Salivary buffering capacity and concentration of 8-isoprostane in all participants (n = 46). The best-fitting curve was plotted with the Loess method [25].

In all participants, the concentration of 8-isoprostane negatively correlated with the buffering capacity (Pearson correlation coefficient *r* = −0.341, *p* = 0.032, Spearman’s *rho* = −0.169 *p* = 0.297), which was mainly evident for 8-isoprostane values >200 pg/mL (Figure 1). Thus, high concentrations of 8-isoprostane were found in the saliva samples with a low buffering capacity. Most of the samples exhibited good buffering capacity (final pH > 6.0 after acid addition) and low concentrations (≤300 pg/mL) of 8-isoprostane.

The salivary concentrations of 8-isoprostane before and after the dental treatment exhibited a positive correlation in the caries-active group (Pearson correlation coefficient r = 0.461, *p* = 0.041, Spearman’s *rho* = 0.544, *p* = 0.013).

## 4. Discussion

Based on the research conducted for this study, this is the only study that has utilized 8-isoprostane as a salivary biomarker of the oxidative stress level in relation to dental caries and treatment. The present results revealed significant differences in 8-isoprostane levels before and after dental restorative treatment. Significant differences have also been reported in the salivary characteristics between caries-active and caries-free children. Among the factors examined, the salivary flow rate and the concentration of 8-isoprostane did not relate to caries activity; however, the latter significantly decreased after the restorative treatment.

The oxidant/antioxidant status of saliva has earlier been examined with various stress indicators in relation to dental caries. The TAC of saliva was elevated in children with early childhood caries [10,15,19,26,27,28,29]. Similarly, higher concentrations of MDA were observed in the saliva of caries-active children compared to caries-free children [7,8,17]. Other studies showed a tendency to increase oxidative stress, but no statistically significant differences were found [7,30]. The present study shows no correlation between caries activity and oxidative stress using 8-isoprostane in saliva as the biomarker. The variation in our study’s results, compared to those mentioned earlier, stems from the use of a different oxidative stress marker.

The choice of 8-isoprostane was based on the compound’s advantages compared to other biomarkers and, additionally, its extensive use for oxidative stress assessment in human studies [31,32,33]. Isoprostanes are stable substances that exist at detectable levels in all biological fluids and can easily be estimated [33]. They are produced as end products upon the peroxidation of lipids in membranes and lipoproteins, with these compounds being major peroxidation targets in the organism [34,35,36].

The salivary levels of 8-isoprostane have been studied in relation to other oral conditions and found to increase with diseases affecting soft oral tissues, such as lichen planus, squamous cell carcinoma, gingivitis, chronic periodontitis, and oral submucous fibrosis [11,13,37,38,39]. However, there has been no previous study on 8-isoprostane as a biomarker for caries status.

It may be argued whether the lack of an association between caries activity and 8-isoprostane levels, as found presently, depends on the biomarker or on other health conditions of the two groups. Concerning the former, one can speculate that this biomarker may be influenced by some oral factors to a greater extent than caries, and, as such, it is not suitable. To address this issue, comparative studies with various biomarkers for oxidative stress must be performed.

When dealing with the latter, it is plausible to assume that several extrinsic and intrinsic factors affecting the oral condition, either by local or systemic actions, may influence the isoprostane levels. Obviously, carious lesions cannot be a source of isoprostane as long as the lesion is limited to the acellular hard dental tissue. Nevertheless, the lesion, especially in its cavitated form, contains a considerable microbial load that is metabolically active and produces toxic substances for the host tissues [38], thus inducing local inflammatory reactions in adjacent soft tissues and indirectly triggering isoprostane production.

Another factor that affects the isoprostane level is the condition of the gingiva, as mentioned above. Caries-active children have undoubtfully insufficient dental plaque control, which causes the disease to occur upon frequent sugar intake [39,40]. Nevertheless, inadequate tooth cleaning may also exist in caries-free subjects, which results in dental plaque accumulation. Limited sugar intake probably helps to avoid caries but not gingivitis. The periodontal condition of the participants was not recorded in detail and analysis in relation to the 8-isoprostane concentration was not possible; however, most of the children had scant oral hygiene habits, which may have influenced the results.

Oxidative stress biomarkers are assumed to increase already in the pre-clinical stage of a disease because of an unhealthy lifestyle [41]. Both caries and periodontal diseases are complex diseases, i.e., their manifestation and progression depend on the interplay of hereditary and lifestyle factors. Obviously, such factors may influence oxidative stress in oral tissues, which makes the role of separate stress-inducing factors difficult to determine through cross-sectional studies.

Nonetheless, the role of dental caries in modifying the oxidative stress of the oral cavity is evident by the finding of a significantly decreased 8-isoprostane level after the restorative treatment. Similar results have been reported with other stress markers [42,43,44]. The reduction in oxidative stress may be attributed not only to the resolution of caries-related inflammation and the subsequent healing process [45] but also to improved oral hygiene, which helps eliminate dental plaque and reduces inflammation in soft tissues. Additionally, modifications in patients’ dietary habits could affect these outcomes [46]. Diet, as an external factor, plays a critical role in influencing both oxidative damage and antioxidant defense mechanisms, which partly explains its connection to chronic diseases such as atherosclerosis, diabetes, cancer, and dental caries [47]. Both epidemiological and clinical data suggest that excessive body fat and unhealthy dietary habits, particularly high sugar consumption, are associated with elevated oxidative stress [48,49], while an inverse relationship exists between fruit and vegetable consumption and oxidative stress [50].

Contrary to the treatment-associated decrease in oxidative stress observed in caries-active children, another study showed unaffected TAC upon the treatment of dental abscesses [45]. However, these authors only treated the teeth with dental abscesses rather than performing a comprehensive dental treatment, which may explain the unchanged TAC [15].

The concentration of 8-isoprostane negatively correlates with the buffer capacity. This is consistent with the higher levels of MDA and the lower salivary buffer capacity found in caries-active children compared to caries-free children [6]. Furthermore, caries-active children demonstrated significantly lower pH levels and conductivity compared to caries-free children, with these findings also being in agreement with earlier observations [8,27,50]. Concerning the salivary flow rate, the existing evidence remains inconclusive, with some studies suggesting an association between low salivary flow rates and dental caries and other studies, like the present one, reporting the contrary [51,52].

The relation between caries activity and lower levels of salivary conductivity has not been previously reported. Neither has an increase in conductivity been shown after the caries treatment. Salivary conductivity is recognized as an indicator of hydration status, with research indicating that conductivity increases during dehydration and decreases following rehydration [53]. We attempted to diminish the effect of possible dehydration by asking all participants to drink 0.5 l of water 1 h prior to sampling. Increased conductivity also indicates increased ionic strength, which may reduce the demineralization of teeth and favor remineralization and could explain the association with caries. Other factors of importance for the balance of mineralization are the pH and the buffer capacity, which were also improved after the treatment. Similar findings have been reported by others [54,55,56]. These findings underscore the complex relationship and the significant role of the physicochemical properties of saliva in the development of dental caries and the possible favorable effect of caries treatment on salivary qualities as well [8,57].

Concerning the limitations of this study, 8-isoprostane, as an oxidative stress marker, has not been previously investigated in relation to dental caries and treatment. As a result, no formal sample size estimation or power analysis was conducted. Additionally, the sample size of 46 children may limit the generalizability of the findings, and a larger cohort would provide greater statistical power. The pH of the matrix can influence antibody affinity in immunoassays and subsequently impact measured concentrations. This could contribute to some of the correlations seen between saliva biochemistry parameters and the oxidative stress marker measured with the immunoassay in saliva with a variable pH and buffering capacity. Thus, the antigen–antibody binding affinity in the assay could be affected by the pH/buffering capacity of saliva. Moreover, the commercial assay used has not been previously specifically validated for saliva; nevertheless, similar assays have been successfully used in previous saliva-based studies. The salivary flow rate may have also been underestimated due to the young age of the participants and potential challenges in ensuring consistent cooperation during saliva collection. Another consideration is the possible effect of sample storage duration at −80 °C on 8-isoprostane concentration estimation. While all samples were promptly stored at −80 °C to minimize oxidative degradation, we lack statistical data on the relationship between storage times and the observed results. Finally, the study’s short follow-up period restricts the ability to assess long-term changes in physicochemical properties and 8-isoprostane levels following dental treatment.

## 5. Conclusions

Salivary physicochemical properties, such as pH, conductivity, and buffering capacity, were found to be improved upon caries treatment and tended to reach levels closer to the corresponding ones for caries-free children. Moreover, we observed a decreased concentration of the oxidative stress factor 8-isoprostane in saliva following the treatment of caries. The design of our study did not allow us to conclude whether our findings could be attributed to the causal effect of the treatment. Properly designed intervention studies are necessary to confirm the observed associations.

## Figures and Tables

**Table 1:** Characteristics of the study groups. Data are presented as the mean ± standard deviation.

Characteristics	Group	
	Caries-Active	Caries-Free
Age (months)	91.9 ± 22.6	93.9 ± 26.9
Males [number, (%)]	13 (50)	12 (60)
DMFT/dmft index	8.9 ± 4.2	0.0 ± 0.0
DT index	2.1 ± 1.9	0.0 ± 0.0
dt index	6.9 ± 3.7	0.0 ± 0.0
DT + dt index	9.1 ± 4	0.0 ± 0.0

**Table 2:** Salivary characteristics in relation to the caries status. The asterisk indicates statistically significant differences between the two groups (Student’s *t*-test or the Mann–Whitney test was used for independent samples). Data are presented as the mean ± standard deviation (SD) and median (min–max) plus the corresponding effect sizes (absolute values of Cohen’s *d* or *r*).

Characteristics	Group				*p*-Value ^+^	Effect Size
	Caries-Active		Caries-Free			
	Mean ± SD	Median (Min–Max)	Mean ± SD	Median (Min–Max)		
Flow rate (mL/min)	0.54 ± 0.23	0.50 (0.22–1.00)	0.58 ± 0.37	0.50 (0.20–1.70)	0.840	*r* = 0.031
Conductivity (μS/cm)	2255.38 ± 565.09	2140.00 (1200.00–4000.00)	2700.00 ± 655.34	2665.00 (1560.00–4380.00)	0.018 *	*d* = 0.734
pH	7.29 ± 0.52	7.30 (6.10–8.00)	7.85 ± 0.53	7.85 (7.10–9.00)	0.001 *	*d* = 1.071
Buffer capacity (pH)	6.59 ± 0.95	6.70 (3.80–8.00)	7.13 ± 0.83	7.10 (5.20–8.60)	0.070	*r* = 0.267
8-isoprostane (pg/mL)	189.10 ± 186.17	116.25 (5.90–662.00)	205.76 ± 140.96	180.70 (44.90–517.20)	0.311	*r* = 0.163

^+^ For the parameters of conductivity and pH, the *t*-test was performed. For the other three parameters, the Mann–Whitney test was performed.

**Table 3:** The 95% confidence intervals (C.Is.) around the mean values of the examined parameters per group.

Characteristics	Group	
	Caries-Active 95% C.I.	Caries-Free 95% C.I.
Flow rate (mL/min)	0.45–0.64	0.41–0.75
Conductivity (μS/cm)	2027.14–2483.63	2393.29–3006.71
pH	7.08–7.50	7.60–8.10
Buffer capacity (pH)	6.20–6.97	6.74–7.51
8-isoprostane (pg/mL)	101.97–276.23	139.78–271.73

**Table 4:** Salivary characteristics of the caries-active subjects before and after the restorative treatment. The asterisk indicates statistically significant differences between the values before and after treatment (Student’s *t*-test or the Wilcoxon test was for paired samples). Data are presented as the mean ± standard deviation (SD) and median (min–max) plus the corresponding effect sizes (absolute values of Cohen’s *d* or *r*).

Characteristics	Treatment				*p*-Value ^+^	Effect Size
	Before		After			
	Mean ± SD	Median (Min–Max)	Mean ± SD	Median (Min–Max)		
Flow rate (mL/min)	0.54 ± 0.23	0.50 (0.22–1.00)	0.52 ± 0.15	0.48 (0.30–1.00)	0.779	*d* = 0.059
Conductivity (μS/cm)	2255.38 ± 565.09	2140.00 (1200.00–4000.00)	2753.48 ± 784.22	2730.00 (1440.00–4300.00)	0.008 *	*d* = 0.606
pH	7.29 ± 0.52	7.30 (6.10–8.00)	7.57 ± 0.60	7.50 (6.50–8.80)	0.074	*d* = 0.392
Buffer capacity (pH)	6.59 ± 0.95	6.70 (3.80–8.00)	7.49 ± 0.69	7.50 (5.20–8.90)	<0.001 *	*r* = 0.597
8-isoprostane (pg/mL)	189.10 ± 186.17	116.25 (5.90–662.00)	98.70 ± 78.81	76.30 (4.10–290.69)	0.030 *	*r* = 0.336

^+^ For the parameters of conductivity and pH, the *t*-test was performed. For the other three parameters, the Mann–Whitney test was performed.

**Table 5:** The 95% confidence intervals (C.Is.) around the mean values of the examined parameters before and after treatment.

Characteristics	Treatment	
	Before 95% C.I.	After 95% C.I.
Flow rate (mL/min)	0.45–0.64	0.46–0.59
Conductivity (μS/cm)	2027.14–2483.63	2414.35–3092.60
pH	7.08–7.50	7.31–7.82
Buffer capacity (pH)	6.20–6.97	7.19–7.79
8-isoprostane (pg/mL)	101.97–276.23	61.82–135.58

**Table 6:** Correlations between caries activity (expressed as the total number of deciduous and permanent teeth with carious lesions DT+ dt) and salivary characteristics in all participants (*n* = 46). Pearson’s correlation coefficient (*r*) and Spearman’s *rho* are given. The asterisk indicates statistically significant correlations between the examined parameters.

Characteristics	Caries Activity			
	Pearson’s *r* Correlation Coefficient		Spearman’s *rho*	
	*r*	*p*-Value	*rho*	*p*-Value
Flow rate (mL/min)	−0.146	0.338	−0.24	0.874
Conductivity (μS/cm)	−0.390	0.008 *	−0.398	0.007 *
pH	−0.437	0.003 *	−0.467	0.001 *
Buffer capacity (pH)	−0.238	0.115	−0.208	0.170
8-isoprostane (pg/mL)	−0.204	0.206	−0.279	0.081

## Data Availability

The data presented in this study are available on request from the corresponding author. The data are not publicly available due to institutional restrictions.
